# Disturbances in Cholesterol Homeostasis and Non-alcoholic Fatty Liver Diseases

**DOI:** 10.3389/fmed.2020.00467

**Published:** 2020-09-02

**Authors:** Pooja Malhotra, Ravinder K. Gill, Seema Saksena, Waddah A. Alrefai

**Affiliations:** ^1^Division of Gastroenterology and Hepatology, Department of Medicine, University of Illinois at Chicago, Chicago, IL, United States; ^2^Jesse Brown VA Medical Center, Chicago, IL, United States

**Keywords:** NPC1L1, ezetimibe, statins, bile acids, fatty liver

## Abstract

Non-alcoholic fatty liver disease (NAFLD) is a major health problem associated with obesity and other features of the metabolic syndrome including insulin resistance and dyslipidemia. The accumulation of lipids in hepatocytes causes liver damage and triggers inflammation, fibrosis, and cirrhosis. Beside fatty acids and triglycerides, evidence showed an increased accumulation of free cholesterol in the liver with subsequent toxic effects contributing to liver damage. The maintenance of cholesterol homeostasis in the body requires a balance between several pathways responsible for cholesterol synthesis, transport and conversion into bile acids. Intestinal absorption is also one of the major determinants of cholesterol homeostasis. The nature of changes in cholesterol homeostasis associated with NAFLD has been a subject of extensive investigations. In this article, we will attempt to provide a brief overview of the current knowledge about the disturbances in cholesterol metabolism associated with NAFLD and discuss how certain molecular targets of these pathways could be exploited for the treatment of this multifactorial disease.

## Introduction

Non-alcoholic fatty liver disease (NAFLD) is a common chronic hepatic disorder associated with obesity and metabolic syndrome (1, 2). Histologically, NAFLD encompasses a broad range of liver injury including simple steatosis or non-alcoholic fatty liver (NAFL), non-alcoholic steatohepatitis (NASH) and subsequent fibrosis as well as cirrhosis (3). The key feature is lipid accumulation in the liver that is not related to alcohol in hepatocytes (4). The accumulation of lipids in hepatocytes as a result of dyslipidemia and insulin resistance leads to liver damage and triggers elaborate response causing hepatic inflammation and fibrosis (2). It is widely accepted that increased flux of fatty acids associated with insulin resistance and/or increased *de novo* lipogenesis in the liver lead to fatty acid accumulation in the liver with associated lipotoxicity (5). However, emerging evidence also suggested that the increased level of cellular cholesterol contributes to the development of NAFLD and the progression of the disease (6). Understanding the roles of cholesterol in the pathophysiology of NAFLD is of a particular importance as it may unravel novel molecular targets for effective therapeutic interventions for NAFLD. In this review, we will give a brief summary about processes involved in the maintenance of cholesterol homeostasis and discuss their potential roles in NAFLD. A synopsis of current knowledge regarding the benefits of cholesterol lowering drugs in the management of patients with NAFLD is also included.

## Cholesterol Homeostasis

Maintaining cholesterol pool in the body is achieved by balancing between input and output pathways of cholesterol metabolism (7). Components of input pathways include endogenous *de novo* synthesis and intestinal absorption of cholesterol, whereas the output pathways include the excretion of free cholesterol in bile, the conversion into bile acids, and the non-biliary trans-epithelial cholesterol efflux (TICE) in the intestine (8, 9). Cholesterol is secreted into the circulation in the very low-density lipoprotein (VLDL) from the liver, and its clearance from the blood involves the uptake of low-density lipoprotein (LDL) by the LDL receptor (LDLr). Cholesterol is transported from the peripheral tissues to liver in the high-density lipoproteins (HDL) particles by the process of reverse cholesterol transport (8).

**Figure 1:**
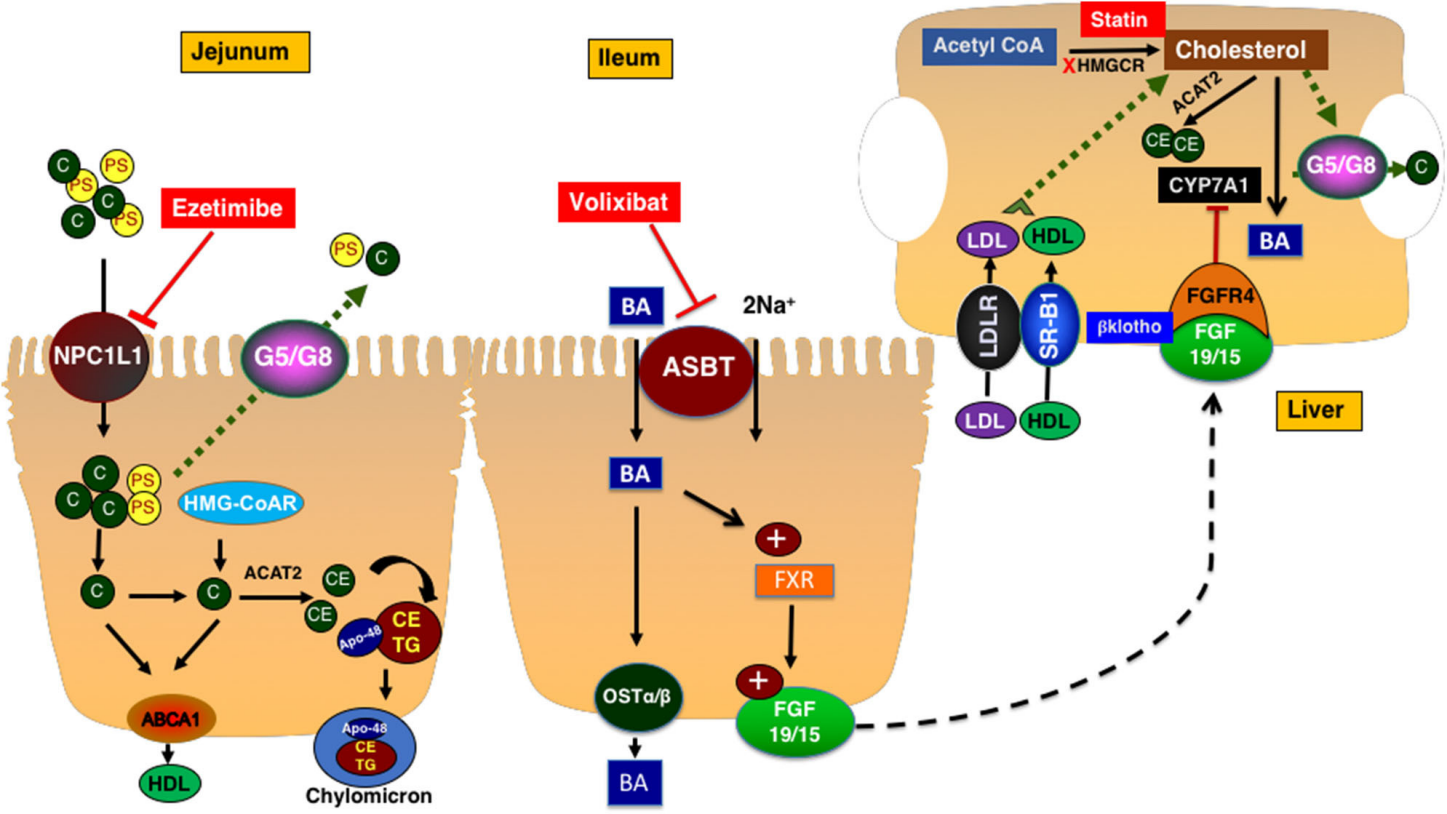
Cholesterol metabolism and transport. NPC1L1 mediates the uptake of cholesterol and plant sterols into small intestinal epithelial cells. The majority of plant sterols are secreted back into the lumen by the action of ABCG5/G8 heterodimer. Cholesterol is esterified and packaged into chylomicrons along with triglycerides. Ezetimibe inhibits NPC1L1 and decreases the intestinal cholesterol absorption. Cholesterol is synthesized in the liver and intestine. ABCA1 transporter mediates the efflux of cholesterol to a nascent HDL particle. Cholesterol enters the hepatocytes via LDLr or the HDL receptor SR-B1. In hepatocytes, cholesterol is esterified, secreted as free cholesterol into bile via ABCG5/G8 transporters, or converted to bile acids with a process in which CYP7A1 mediates the rate-limiting step. Bile acids are reabsorbed by ASBT in the ileum and activate FXR which increased the expression and secretion of FGF19 in humans (15 is rodents). FGF19/15 circulates back to the liver to inhibit CYP7A1 via FGF4 receptor and β-klotho co-receptor. ASBT, apical sodium-dependent bile acid transporter; BA, bile acid; CYP7A1, cytochrome P450 7A1; FGF15/19, fibroblast growth factor 15/19; FXR, farnesoid X receptor; Ost a & β, organic solute transporter alpha and beta; HMGCR, 3-Hydroxy-3-methylglutaryl-CoA Reductase; C, cholesterol; PS, plant sterols; NPC1L1, Niemann-pick C1 Like 1; G5/G8, ATP binding casette transporter G5 and G8; SR-B1, scavenger receptor B1; ABCA1, ATP-binding cassette transporter ABCA1; ACAT2, Acetyl-Coenzyme A Acetyltransferase 2; HDL, High density lipoprotein; CE, cholesterol ester; TG, triglyceride; FGFR4, fibroblast growth factor receptor 4; LDLR, Low density lipoprotein receptor; LDL, low density lipoprotein.

Intestinal cholesterol absorption represents one of the major determinants of cholesterol homeostasis. The total amount of cholesterol in the intestinal lumen is derived from multiple sources including diet (~300–500 mg/day from western-type of diet) as well as the epithelial cells that are shed into the lumen (~300 mg/day) and biliary cholesterol (~800–1,200 mg/day). A total amount of ~1,400–1,700 mg of cholesterol is presented in the intestine on a daily basis for absorption (10). The efficiency of cholesterol absorption exhibits wide inter-individual variations (28–80%) and depends on several luminal (emulsifiers: bile acids, phospholipids) and epithelial factors (10). Cholesterol uptake by enterocytes is mainly mediated by the specific transporter protein Niemann-Pick C1 Like 1 (NPC1L1), which is abundantly expressed on the brush-border membrane of small-intestinal enterocytes (8, 11) (Figure 1). Cholesterol is esterified by acetyl-CoA cholesterol acyltransferase 2 (ACAT-2) and incorporated along with triglycerides and apolipoprotein B-48 into chylomicrons. Triglycerides are hydrolyzed by lipoprotein lipase and chylomicrons are transformed into chylomicron remnants that are taken up by the liver (10).

The *de novo* synthesis of cholesterol occurs in all cells in the body. However, the liver represents the main site for cholesterol synthesis and storage (8). Cholesterol synthesis starts with acetyl-CoA and involves multiple reactions. The two rate-limiting steps in this complex process are mediated by 3-hydroxy-3-methyl-glutaryl CoA reductase (HMGCoAr) and squalene monooxygenase. Cholesterol, the newly synthesized in the liver and the one derived from the intestinal absorption, is then packaged along with triglycerides and apolipoprotein B-100 into the VLDL particles that are assembled by the microsomal transfer protein. The VLDL loses its load of triglycerides while circulating in the tissues by lipoprotein lipase and transforms into LDL that represents the main vehicle of cholesterol transport to the peripheral tissues. Other processes involving cholesterol in the liver include esterification by ACAT2 for storage, excretion into bile by the function of heterodimer of ATP-binding cassette transporters ABCG5/G8 found on the canalicular membrane of hepatocytes, or the conversion to bile acids by a complex process in which the cytochrome P450 7A1 (CYP7A1) enzyme mediates the rate-limiting reaction (8). The expression of CYP7A1 is mainly regulated by a signal generated from the ileum represented by fibroblast growth factor 19 in human and 15 in rodents (FGF19/15) (12). The synthesis and secretion of FGF19/15 are induced by the uptake of bile acids by enterocytes in the distal ileum that is mediated by the action of the apical sodium dependent bile acid transporter ASBT. The inhibition of ASBT reduces FGF19/15 expression and promotes hepatic bile acid synthesis and therefore, decreases levels of hepatic cholesterol (Figure 1).

The transport of cholesterol from the peripheral tissues back to liver is initiated by cholesterol efflux from cells via the ATP-binding cassette transporter ABCA1 to the nascent apolipoprotein A containing HDL particles. Cholesterol could be transferred from HDL in the circulation to LDL by cholesterol-ester transfer protein (CETP). HDL is taken up by hepatocytes via the scavenger receptor B1 (SR-B1) (8) (Figure 1).

The expression of genes responsible for cholesterol transport and metabolism are tightly regulated by coordinated actions of transcription factors. For example, the decrease in the level of cellular cholesterol activates the ER membrane-bound transcription factor Sterol regulatory element-binding protein isoform 2 (SREBP-2) that induces the expression of HMGCoAr and LDLr (8). On the other hand, the increase in cellular cholesterol elevates the levels of the oxygenated cholesterol intermediates oxysterols that trigger Liver X receptors (LXRs) transcription factors to stimulate the pathways of cholesterol efflux and to promote cholesterol elimination from the liver (8).

## Cholesterol and the Pathophysiology of NAFLD

The interest in understanding the relationship between cholesterol metabolism and the development of NAFLD and NASH was incited by observational studies linking liver damage with the risk for the development of cardiovascular disease (CVD) (13). In fact, the increased prevalence of metabolic syndrome in patients with NAFLD was to such an extent that some authors suggested that NALFD represents the hepatic manifestation in a spectrum of metabolic disorders (14). The metabolic syndrome refers to a cluster of metabolic disturbances including obesity, insulin resistance, hypertension, and atherogenic dyslipidemia that increase the risk for cardiovascular disease, stroke and diabetes mellitus (14). Meta-analysis of observational retrospective and prospective studies demonstrated an increased risk for CVD of both fatal and non-fatal events of CVD in patients with NAFLD as compared to patients without NAFLD (15). Such a link between NAFLD and CVD provided a compelling rationale to closely examine cholesterol metabolism in patients with NAFLD. The features of dyslipidemia associated with NALFD include hypertriglyceridemia and a decrease in HDL cholesterol (2). Although there is no evidence for an increase in total LDL cholesterol, patients with NAFLD were shown to have elevated levels of highly atherogenic subpopulations of LDL such as the oxidized particles (2). Furthermore, feeding high cholesterol atherogenic diets was able to induce lesions of early NAFLD as well as atherosclerosis in several animal models.

It should be noted that animal species have remarkable differences in cholesterol metabolism and respond differently to cholesterol rich diet. In this regard, it is well-established that rabbits have features of cholesterol metabolism that are closer to those in humans (16). For example, the predominant plasma lipoproteins in both rabbits and humans is LDL whereas it the HDL in mice. This difference is due to the fact that the CETP is lacking in the plasma of mice (17). More importantly, rabbits are sensitive to dietary cholesterol and develop severe hypercholesterolemia in response to high dietary cholesterol with prominent atherosclerosis (16). A recent study showed that diet-induced hypercholesterolemia in rabbits fed with diet containing 1% cholesterol caused liver injury that resembles early lesions of NAFLD (changes in fatty acid and sporadic fibrosis) concomitant with the development of atherosclerosis in the aorta (18). This study generated evidence linking blood cholesterol with NAFLD and provided novel insights into the roles of free cholesterol in inducing liver damage. In the mouse model, hypercholesterolemia and atherosclerosis can be produced only in genetically modified animals such as apoE and LDLr knockout mice (19). In this regard, steatohepatitis was shown in apoE knockout mice in response to high fat high cholesterol feeding (20). Combined transcriptomics and metabolomics analysis demonstrated that high dose of dietary cholesterol in apoE knockout mice triggered hepatic inflammation (21).

An interesting observation was made in wildtype mice (but not in the transgenic mice models of hypercholesterolemia) showing that high cholesterol in the diet was essential to elicit steatohepatitis and fibrosis after 30 weeks of feeding, whereas high fat diet alone failed to produce the same degree of liver injury (22). This role of high dose of dietary cholesterol in the induction of liver damage in wildtype mice was also illustrated in other species. For example, a study showed that feeding Ossabaw pigs atherogenic diet containing high fat along with 2% cholesterol caused severe metabolic syndrome along with lesions in the liver with features resembling human steatohepatitis including microvesicular and macrovesicular steatosis, fatty Kupffer cells, hepatocyte ballooning and fibrosis (23). Collectively, the evidence from preclinical animal models strongly indicate that a high dose of cholesterol is an independent risk factor for liver damage and the development of atherosclerosis. A conclusion can be drawn from these studies linking NAFLD with atherosclerosis implicating the accumulation of cholesterol in the liver and arteries as a trigger for inflammation and subsequent manifestations of liver disease and CVD.

Beside the studies in animal models, the results of a landmark study in humans also supported the role of cholesterol in NAFLD/NASH. Puri et al. (24) showed high levels of free cholesterol in patients with NAFLD (simple steatosis) that were more pronounced in cases with progressive form of the disease, NASH. Cholesterol esters remained unchanged despite the increase in free cholesterol suggesting an impaired function and/or expression of the enzyme responsible for cholesterol esterification (ACAT). Collectively, the evidence from animal models and humans indicates that cholesterol is one of the toxic lipids that accumulates in the liver contributing to liver damage during the course of NAFLD and the progression to NASH.

Recent studies investigated the molecular mechanisms underlying the toxic effects of high cholesterol in the liver. The findings of Mari et al. (25) provided evidence showing that accumulation of free cholesterol but not triglycerides or fatty acids makes hepatocytes more sensitive to TNFa and Fas-mediated apoptosis due to depletion of mitochondrial glutathione. Other studies suggested that the accumulated free cholesterol in damaged hepatocytes precipitates as crystals which in turn induce inflammatory response by interacting with NLRP3 inflammasomes of Kupffer cells (26). Free cholesterol was shown also to directly accumulates in lysosomes of Kupffer cells triggering an inflammatory response (27). Such inflammatory cascade was blocked by 27-hyroxycholesterol that prevented the accumulation of cholesterol in Kupffer cells and reduced NASH. The accumulation of cholesterol in hepatic stellate cells (HSCs) was also implicated in the progression to NASH and fibrosis (28). For example, studies provided evidence showing that the accumulation of free cholesterol in HSCs resulted in an increase in the expression of TLR4 receptor and an increase in the sensitivity of HSCs to TGFb with subsequent fibrosis (29). It is apparent therefore, that increased cellular levels of cholesterol in different cell types in the liver may be responsible for liver damage and the progression from simple steatosis to NASH.

## Molecular Pathways Underlying Cholesterol Accumulation in NAFLD

The accumulation of free cholesterol in NAFLD may occur due to an upregulation in the input pathways or a decrease in the elimination of cholesterol. Studies showed that the expression of HMGCoAr are increased in patients with NAFLD/NASH (30). There is evidence to suggest that changes in microRNAs (miRs) might be responsible for the observed increase in HMGCoAr. Several studies demonstrated the roles of several miRs in the development of liver diseases including NAFLD. The roles of these miRs including miR-122, miR-33, and miR-24a are discussed in details in other excellent review articles (31, 32). Studies have also demonstrated the regulation of several genes involved in cholesterol metabolism such as HMGCoAr by miRs and these findings are nicely summarized in other review articles (33, 34).

With respect to cholesterol metabolism as it relates to NAFLD/NASH, miR-34a levels were shown to be elevated in patients with NAFLD/NASH causing a decrease in hepatic NAD-dependent deacetylase Sirtuin1 with subsequent dephosphorylation and an increase in HMGCoAr (30). The roles of miR-29a was also suggested by a supporting evidence showing a negative correlation between miR-29a and HMGCoAr expression after 3 weeks feeding of methionine-choline deficient diet (MCD) to mice (35). The study revealed that 3′ un-translated region (3′UTR) of HMGCoAr was targeted by miR-29a (35). The decrease in miR-29a was concomitant with a decrease in hepatic Dicer1 enzyme that is essential for the maturation of microRNAs. In fact, liver-specific knockout of Dicer1 enzyme resulted in an increase in HMGCoAr associated with accumulation of free cholesterol in the liver (35). These findings revealed an important molecular pathway involving hepatic Dicer1 and microRNAs in the increase in cholesterol synthesis and accumulation of free cholesterol in the liver contributing to the development of NAFLD/NASH.

The expression of SREBP2 transcription factor was also increased in patients with NAFLD/NASH (30). Since SREBP2 induces the expression HMGCoAr, the observed increase in active SERBP2 represents an additional molecular pathway that might be responsible for the increases in hepatic levels of free cholesterol. Mari et al. (25) demonstrated that SREBP2 overexpression was not found in obese patients and patients with other chronic liver diseases such as hepatitis C, but was rather a specific feature associated with NAFLD/NASH. It should be noted that the accumulation of free cholesterol in hepatocytes is expected to inhibit the activation of SREBP2 (36). It is possible that SREBP2 is activated in NAFLD/NASH by a mechanism independent from the canonical pathway related to changes in cellular cholesterol (37). Indeed, a previous study showed that the injection of cytokines in C57BL mice resulted in an increase in hepatic SREBP2 and HMGCoAr expression (38). Similar findings were obtained in response to incubation of human hepatic HepG2 cells with cytokines. Although loading of HepG2 cells with cholesterol inhibited the SREBP2 pathways as expected, such a negative feedback inhibition was overridden by inflammatory stress caused by the cytokines (38). It is also reported that miR-122 stabilizes the inactive form of SREBP2 (39). Interestingly, miR-122 was shown to be significantly decreased in patients with NASH, thus providing a potential explanation for the increase in SREBP2 (39). It is possible also that hyperinsulinemia resulted from increased insulin resistance may also cause an increase in the active form of SREBP2 (40). Collectively, the inflammatory stress, the decrease in miR-122 and associated hyperinsulinemia in NASH presents a reasonable explanation for the constant activation of SREBP2 bypassing the canonical negative feedback inhibitory effect of high levels of cellular cholesterol (37). Beside the role of hepatic SREBP2 in the pathophysiology of NAFLD/NASH, our studies in transgenic mouse model of intestine-specific overactivation of SREBP2, demonstrated hepatic steatosis, and increased susceptibility of the animals to severe liver damage and profound inflammation and fibrosis in response to a high fat/high cholesterol diet (41). These observations strongly suggest that the activation of SREBP2 not only in the liver but also in the intestine plays a critical role in the development of NAFLD/NASH.

Beside the increase in cholesterol synthesis, changes in the pathways involved in the elimination of cholesterol were also noted in patients with NASH. Among these observations is the decrease in the expression of CYP7A1 implicating reduced bile acid synthesis from cholesterol (30). Also, a decrease in the expression of cholesterol transporters ABCG5/G8 responsible for cholesterol excretion into bile was reported (30). It is clear therefore, that the accumulation of hepatic free cholesterol in NAFLD/NASH patients is multifactorial with underlying increase in synthesis and decrease in the elimination of cholesterol.

With respect to dietary cholesterol and liver disease in humans, an epidemiological study suggested that dietary cholesterol is an independent risk factor for liver cirrhosis and hepatic cancer (42). These studies provide support to the observations made in animal models demonstrating the role of cholesterol in the induction of liver damage (43, 44). Since dietary cholesterol represents only a small portion of the total amount of luminal cholesterol presented for absorption (10), one could argue that the relation between the efficiency of cholesterol absorption and the development of liver diseases including NALFD/NASH should be carefully investigated. One study by Simonen et al. (45) evaluated serum surrogate markers for cholesterol synthesis (cholestenol, desmosterol, and lathosterol) and absorption (plant sterols). Individuals with NALFD were identified as those having liver fat ≥ 5.56% as judged by with proton magnetic resonance spectroscopy. The authors found that the content of liver fat was positively correlated with markers of cholesterol synthesis and inversely correlated with markers of cholesterol absorption (45). It is possible that there is an interrelationship between these two processes so that the decrease in cholesterol absorption may represent a homeostatic response to an increase in cholesterol synthesis (45). It will be important to investigate a possible difference in the efficiency of cholesterol absorption in patients with NASH as compared to those with NAFLD. With respect to the expression of intestinal NPC1L1, previous studies showed an increase in the expression of this intestinal in patients with diabetes mellitus (46). It is of central importance to investigate the expression of NPC1L1 in patients with NAFLD and NASH to a make a conclusion regarding potential changes in cholesterol absorption. Also, the studies mentioned above assessed the efficiency of cholesterol absorption indirectly by measuring serum plant sterols as surrogate markers. A direct measurement of cholesterol absorption in patients with NAFLD/NASH is warranted.

## Cholesterol Lowering Drugs in the Management of NAFLD/NASH

According to current guidelines, decreasing the levels of plasma cholesterol to stringent low levels is highly recommended in patients with high risk for CVD (47, 48). Patients with NAFLD/NASH have high risk of developing CVD (15, 49). Therefore, it is of central importance to address treatment options available to lower plasma cholesterol in this patient population. Table 1 summarizes the results of selected studies related to cholesterol-based therapy for NAFLD.

**Table 1:** Therapeutic interventions for NAFLD/NASH.

**Therapeutic intervention**	**Therapeutic agent**	**Molecular target**	**Effect on NALFD/NASH**	**Model**	**Ref**
Inhibition of cholesterol synthesis	Statins	HMGCoAr	Protection against steatosis, steatohepatitis and fibrosis [assessed by liver biopsies]	Retrospective study-humans	(50)
	Simvastatin	HMGCoAr	Protects parenchymal and endothelial components of the liver after warm reperfusion.	Rat model of steatotic graft	(51)
	Fluvastatin	HMGCoAr	Reduced hepatic steatosis and fibrosis scores, α-SMA protein expression, mRNA expression of pro-inflammatory, and pro-fibrogenic genes	Choline-deficient L-amino acid-defined diet-induced Rat NASH model	(52)
	Simvastatin	RhoA and Ras signaling	Decreased hepatic inflammation and fibrosis. No effect on steatosis	Diet-induced NASH in apoE^−/−^ mice	(53)
Inhibition of bile acid absorption	SC-435	ASBT	Restored glucose tolerance, reduced hepatic triglyceride and total cholesterol, improved NAFLD activity score	High fat diet-induced NAFLD in C57BL mice	(54)
	Volixibat	ASBT	Attenuated the hepatocyte hypertrophy, reduced hepatic triglyceride and cholesteryl ester levels, decreased NAFLD activity score	Diet-induced NASH in LDLr–/–/ Leiden mice	(55)
	Volixibat	ASBT	Interim-endpoint was not met and the study was terminated owing to lack of efficacy	Clinical trial-humans	(56)
Inhibition of cholesterol absorption	Ezetimibe	NPC1L1	Decreased hepatic cholesterol content and increased the hepatic total bile acid content, ameliorated hepatic insulin resistance	C57BL mice fed with high fat diet	(57)
	Ezetimibe	NPC1L1	Fibrosis stage and ballooning score were significantly improved with ezetimibe treatment [Histological evaluation]	Clinical trial-humans	(58)
	Ezetimibe	NPC1L1	Failed to reduce liver fat assessed by MRI-PDFF imaging	Clinical trial-humans	(59)
	Ezetimibe	NPC1L1	Decreased NAFLD activity score (NAS) but not hepatic steatosis	Meta-analysis of RCTs-humans	(60)

### Inhibitors of Cholesterol Synthesis

Statins, the inhibitors of HMGCoAr, are the widely used drugs to lower plasma cholesterol (61). It is well-established that statins reduce LDL cholesterol and significantly decrease the risk for CVD. Thus, it is logical to use statins in NAFLD patients who are at risk for CVD (62). The use of statins was noted to be associated with an increase in the levels of serum transaminases raising concerns about their safety especially in patients with NAFLD/NASH (63). However, recent studies in a cohort of ~1,200 European patients provided strong evidence demonstrating the beneficial effects of statins in the protection against steatosis, steatohepatitis, and fibrosis as assessed by liver biopsies (50). Several other meta-analysis of available data suggested that atorvastatin improved liver injury in NAFLD/NASH patients and was more potent in decreasing CVD risk as compared to individuals with normal liver function (64).

The effects of statins on several aspects of liver injury was shown in animal models (51). Statins were shown to decrease experimentally induced fibrosis in rats by inhibiting the activation of hepatic stellate cells (52). Recent study also showed that simvastatin reduced liver inflammation and fibrosis in ApoE knockout mice fed with western-type of diet (53). These effects were attributed to the inhibition of RhoA and Ras signaling (53). It is possible therefore, that the amelioration of liver injury by statins is due to other effects beside the inhibition of cholesterol synthesis (65). Statins may also affect cholesterol metabolism in other organs and indirectly influence liver function. For example, it was shown that statin treatment in humans increased the expression of intestinal SREBP2 (66). In this regard, our studies demonstrated that the intestine-specific overexpression of SREBP2 caused hepatic steatosis and profound diet-induced liver injury in mice (41). It is possible therefore, that statins may negatively affect liver function by increasing intestinal SREBP2 expression. In light of the complexity underlying the global effects of statins, comprehensive studies are warranted to delineate the molecular pathways involved in the effects of statins on NAFLD/NASH.

### Inhibitors of Bile Acid Absorption

Blocking the absorption of bile acids eliminates the negative feedback inhibition on their hepatic synthesis and promotes cholesterol degradation (12). ASBT mediates the first and rate limiting step in bile acid absorption and its inhibition represents an attractive therapeutic target to reduce hepatic cholesterol toxicity in NAFLD/NASH (12). Recent studies provided a compelling evidence showing that the lack of ASBT in knockout mice protected against diet-induced liver injury and steatosis (54). Further, these studies showed that the pharmacological inhibition of ASBT significantly decreased hepatic triglycerides and total cholesterol and improved NAFLD activity score (54). Additional studies using the ASBT inhibitor volixibat in LDLr leiden knockout mice supported that conclusion and demonstrated a significant decrease in diet-induced Non-alcoholic fatty liver disease activity score by the inhibition of ASBT (55). In humans however, a phase 2 clinical trial to assess the effects of 48 weeks treatment with the ASBT inhibitor, volixibat, was terminated after mid-term assessment due to lack of improvement in NASH score as judged by MRI-PDFF imaging (56). It should be noted that the histological evaluations including the scores for ballooning and inflammation in this clinical trial were lacking. Such histological analysis in response to treatment with volixibat is crucial as it may provide more insights on the potential effects of inhibiting bile acid absorption on liver injury in NAFLD/NASH. Also, additional studies in preclinical models seem essential to unravel the molecular basis for the observed improvement of NAFLD/NASH in the animal models. Enhanced understanding of these pathways at the molecular level may unravel novel targets that could be more efficacious in the treatment of NALFD/NASH in humans.

### Inhibitors of Cholesterol Absorption

Studies showed that the inhibitor of cholesterol absorption ezetimibe reduces plasma cholesterol and decreases the risk of CVD when used alone or in combination with statins (67, 68). Earlier studies in animal models showed that ezetimibe alleviated liver steatosis in mice fed with high fat diet and had promising effects on NAFLD/NASH (57). However, the studies about the beneficial effects of ezetimibe in patients with NAFLD/NASH generated conflicting results (69). Takeshita et al. (58) showed in a randomized clinical trial in a cohort of 32 patients with NAFLD that ezetimibe improved hepatic fibrosis. However, the clinical trial by Loomba et al. (59) with 50 patients showed that ezetimibe failed to significantly reduce liver fat in NAFLD as assessed by MRI-PDDF imaging. A recent meta-analysis, which exclusively included studies assessing NAFLD/NASH based on biopsy, with only one study that was based MRI-PDFF imaging, showed that ezetimibe decreased NAFLD activity score (NAS) but not hepatic steatosis (60). It was noted that current available data about ezetimibe and NAFLD/NASH were generated from limited number of clinical trials with small sample size (69). Large scale randomized clinical trials are needed for better assessment of the outcome of ezetimibe on the disease activity in patients with NAFLD/NASH. It is noteworthy to mention that the inter-individual variations in the efficiency in cholesterol absorption may influence the results of these studies. We suggest that this potential confounding factor be taken into consideration when assessing the effects of ezetimibe on NAFLD/NASH.

## Conclusion

Disturbances in cholesterol metabolism contribute to the pathophysiology of NAFLD/NASH. An increase in cholesterol synthesis and a decrease in the pathways responsible for the elimination of cholesterol lead to accumulation of free cholesterol in the liver. The toxicity of high cellular levels of cholesterol in hepatocytes is one of the major factors causing inflammation and fibrosis leading to liver damage. The pathways involved in cholesterol metabolism are potential targets for the treatment of NAFLD/NASH. One challenging area is the complex nature of the processes involved in cholesterol homeostasis. For example, cholesterol absorption exhibits wide interindividual variations that may explain the conflicting results when evaluating the efficacy of cholesterol lowering drugs in the treatment of NAFLD/NASH. Such variations evoke the need for precision medicine approaches and individualized treatment. It is possible that individuals with naturally occurring low efficiency of cholesterol absorption may not benefit from the use of cholesterol absorption blocker ezetimibe. It will be interesting in future studies to stratify patients into relevant subgroups in a rigorous manner and take into consideration the inter-individual variations in cholesterol metabolism when assessing the outcome of cholesterol lowering drugs in the treatment of NAFLD/NASH. Precision medicine approaches necessitate the development of novel and simple methods to directly measure dynamic processes such as cholesterol absorption when individualized therapeutic interventions are to be considered for NAFLD/NASH treatment.

## Author Contributions

WA and PM wrote the initial draft. RG and SS edited the initial manuscript. PM and RG made the figure. RG and WA made the table. PM, RG, SS, and WA edited and approved the final draft. All authors contributed to the article and approved the submitted version.

## Conflict of Interest

The authors declare that the research was conducted in the absence of any commercial or financial relationships that could be construed as a potential conflict of interest.
